# Supplementary material for: Resveratrol, Curcumin and Piperine Alter Human Glyoxalase 1 in MCF-7 Breast Cancer Cells
Source: Int J Mol Sci. 2020 Jul 24;21(15):5244. doi: 10.3390/ijms21155244 (PMC7432303; doi:10.3390/ijms21155244)
Supplement: Supplementary file 1 [file ijms-21-05244-s001.pdf]

Supplementary Material

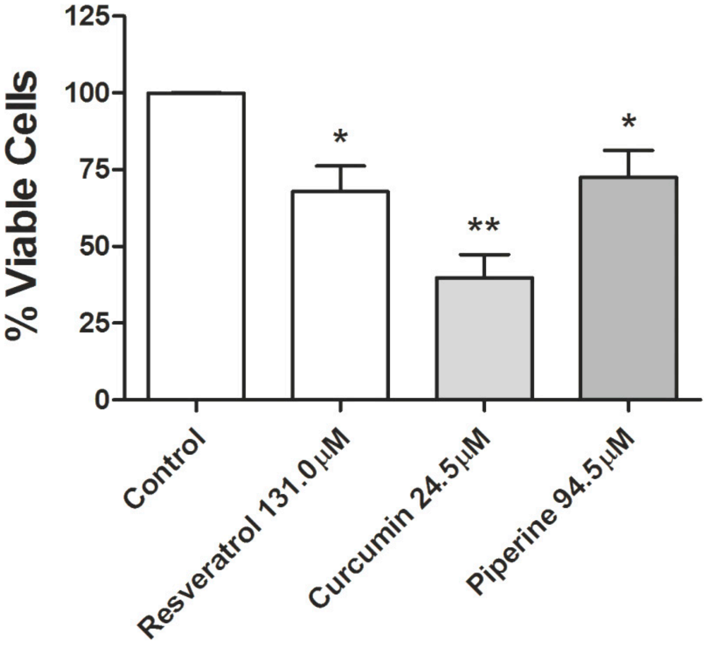

Supplementary Figure 1. Effects of resveratrol, curcumin and piperine on the cell viability of MCF-7 cells. Cells were treated for 24 hours with 131.0 µM resveratrol, 24.5 µM curcumin or 94.5 µM piperine, and the cell viability was then determined by trypan blue dye exclusion assay. The results are representative of three experiments performed in triplicate ± SEM. * $p < 0.05$, ** $p < 0.001$, in relation of control.

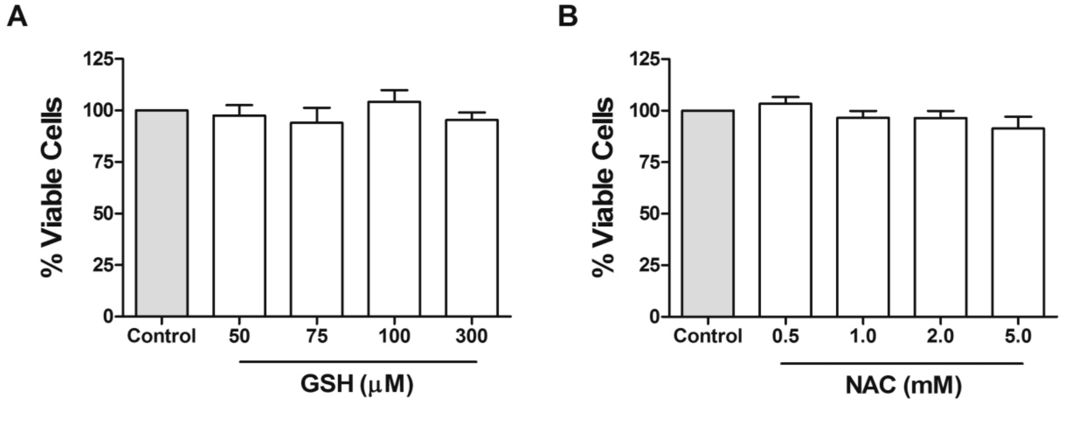

Supplementary Figure 2. Effects of GSH and NAC on the cell viability of MCF-7 cells. Cells were treated for 24 hours with 50.0, 75.0, 100.0 and 300 µM GSH (**A**) or 0.5, 1.0, 2.0, 5.0 mM NAC (**B**), and the cell viability was then determined by MTT assay. The results are representative of three experiments performed in triplicate ± SEM.

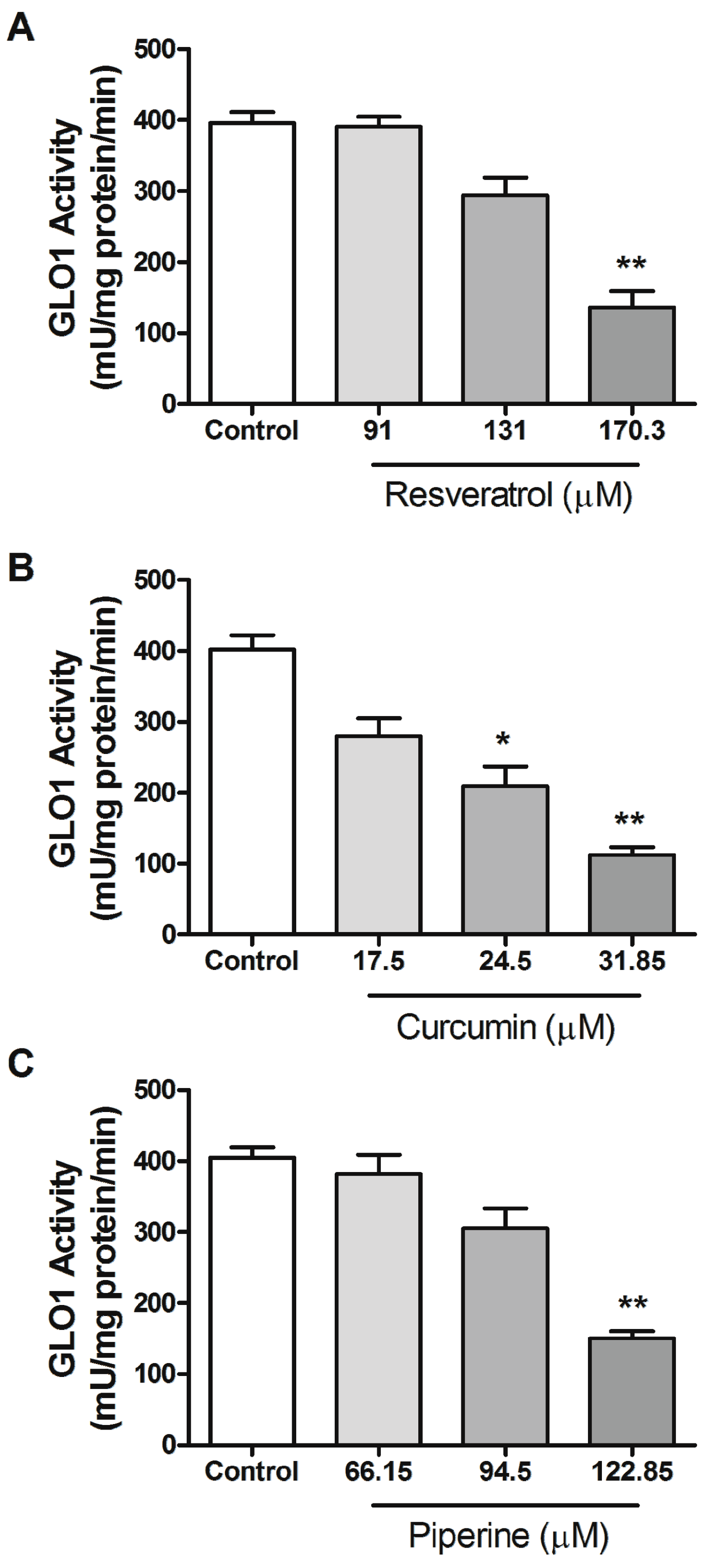

Supplementary Figure 3. GLO1 activity in MCF-7 cells after treatment of resveratrol, curcumin and piperine. Cells were treated with 91.0, 131.0 and 170.3 μM resveratrol, 17.5, 24.5 and 31.85 μM curcumin or 66.15, 94.5 and 122.85 μM piperine. After 24 hours, the GLO1 activity was measured in the extracts of lysed cells. The results are representative of three experiments performed in triplicate ± SEM. * $p < 0.05$, ** $p < 0.001$, in relation of control.

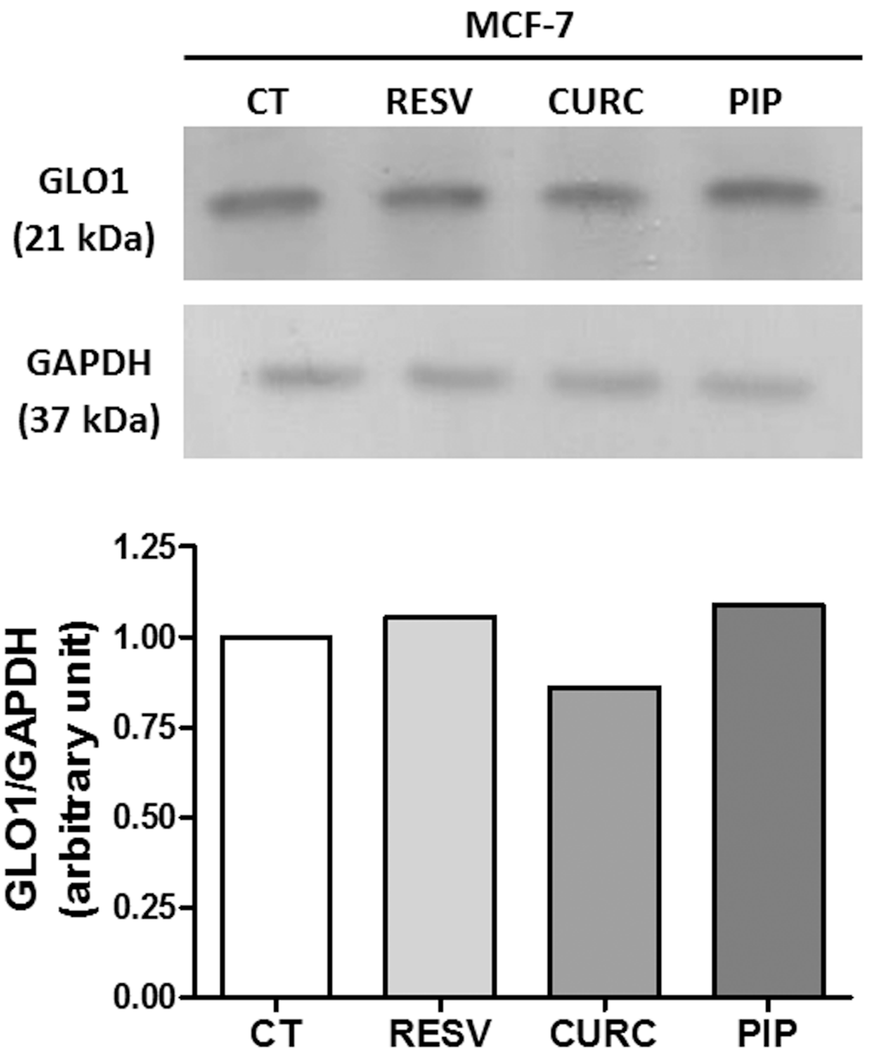

Supplementary Figure 4. Resveratrol, curcumin and piperine effect on GLO1 in MCF-7 cells at the protein level. MCF-7 cells after resveratrol (RESV), curcumin (CURC) and piperine (PIP) treatment with IC_{50} values, respectively, were submitted to Western blot analysis under denaturing conditions. The values were expressed in arbitrary units.
